# Prevalence and antibiotics susceptibility profiles of *Streptococcus pyogenes* among pediatric patients with acute pharyngitis at Felege Hiwot Comprehensive Specialized Hospital, Northwest Ethiopia

**DOI:** 10.1186/s12866-021-02196-0

**Published:** 2021-05-03

**Authors:** Destaw Kebede, Alemale Admas, Daniel Mekonnen

**Affiliations:** 1 Shegaw Motta General Hospital, East Gojjam Zone, Motta Town, Ethiopia; 2 Department of Medical laboratory Science, College of Medicine and Health Sciences, Bahir Dar University, P.O.Box: 79, Bahir Dar, Ethiopia; 3 Biotechnology Research Institute, Bahir Dar University, Bahir Dar, Ethiopia

**Keywords:** Prevalence, *S. pyogenes*, Pediatrics, Acute pharyngitis, Antibiotics susceptibility test

## Abstract

**Background:**

*Streptococcus pyogenes* (*S. pyogenes*) is a Gram positive bacterium which is a leading cause of pharyngitis, skin and soft tissue infection and post streptococcal syndromes. Due to lack of β-lactamase enzyme production, it was considered universally susceptible to penicillin group and later generation of β-lactam antibiotics. As such, empirical treatment was common which might leads to development of antibiotics resistance. Therefore, the aims of this study were to determine the prevalence, antibiotics susceptibility profile; and associated factors of *S. pyogenes* among pediatric patients with acute pharyngitis in Felege Hiwot Comprehensive Specialized Hospital (FHCSH), Northwest Ethiopia.

**Methods:**

Hospital based cross-sectional study was carried out on 154 pediatric patients, whose age ranged from 0 to 18 years old using consecutive convenient sampling technique from 1st February to 19th June 2020 at FHCSH. *S. pyogenes* were identified by throat swab culture on 5% sheep blood agar with an overnight incubation at 37 °C in candle jar containing 5% CO_2_. Gram stain, catalase test and bacitracin test were used to identify *S. pyogenes*. Then,the data were entered into EpiData version 3.1 and analyzed by SPSS version 20 software. Finally, stepwise, bivariable and multivariable logistic regressions were carried out for identifyying factors having significant ssociation (p<0.05) with  acute pharyngitis.

**Results:**

From the total throat swabs, 14 (9.1%) with (95% CI; 4.5–14.3) were culture positive for *S. pyogenes.* From these, all isolates were sensitive to penicillin and ampicillin. On the otherhand, 4 (35.7%), 4 (35.5%), 3 (21.4%), 2 (14.3%), 1 (7.1%), 7 (50.0%) and 1 (7.1%) isolates were resistant for ceftriaxone, vancomycin, erythromycin, tetracycline, chloramphenicol, clindamycin and levofloxacin, respectively. The presence of any smoker in home showed significant association with *S. pyogenes* acute pharyngitis. Furthermore, having tender lymphadenopathy and recurrence were clinical predictors for *S. pyogenes* acute pharyngitis (*P* < 0.05).

**Conclusion:**

The prevalence of *S. pyogenes* was guaged at 9.1% which is considered as low prevalence. All *S. pyogenes* isolats remain sensitive to penicillin. However, resistance was reported to clindamycin 7 (50.0%), ceftriaxone 5 (35.7%) and erythromycin 3 (21.4%). The current practice of giving erythromycin, clindamycin instead of penicillin and ampicillin is againest the microbiology result. Therefore, current empirical treatment of acute pharyngitis shall take in to account the current evidences. Continuous surveillance of antibiotics resistance pattern of *S. pyogenes* for acute pharyngitis must be strengthen to improve the use of antibiotics in hospitals.

## Background

*Streptococcus pyogenes* (*S. pyogenes*) is a Gram positive, extracellular, spherical shape and β-hemolytic bacterium which can grow on enrichment culture media [1]. It was identified as the cause of erysipelas in 1883 by Friedrich Fehleisen [2]. However, in 1933, Rebecca Lancefield made serologic classification of Group A Streptococcus (GAS) based on group A carbohydrate that composed of N-acetyl glucosamine linked to *S. pyogenes* cell wall antigens as rhamnose polymer backbone. As the result, GAS was named as *S. pyogenes* [3].

*S. pyogenes* were responsible for several clinical conditions such as scarlet fever, acute rheumatic fever, glomerulonephritis, sepsis, necrotizing fasciitis, meningitis, streptococcal toxic shock syndrome, impetigo and acute pharyngitis [4]. Acute pharyngitis is one of the disease caused by *S. pyogenes.* It is an inflammation of oropharynx mucous membranes or posterior pharynx and tonsils [5] with different clinical manifestations such as sore throat, sudden onset fever, red pharynx, enlarged tonsils, yellow or blood-tinged exudates, petechiae on the soft palate and posterior pharynx [6].

About hundred millions people develop serious *S. pyogenes* infection every year. It cause about 660,000 invasive infections and 616 million cases of pharyngitis that result in 163,000 death from 2009 to 2014 [7]. In African countries, *S. pyogenes* was isolated from children with acute pharyngitis and the its' prevalence was as high as 66.7, 28, 2.3, and 11.3% in Nigeria [8], Egypt [9], Kenya [10] and Jimma, Ethiopia [11], respectively.

S.yogen transmission can be through direct contact, contaminated fomites, or food borne contamination or droplets from those with pharyngeal infection or colonization [12]. Most *S. pyogenes* infections were treated with penicillin and still being effectively used for emprical treatment [13]. However, those patients with allergic to penicillin have been treated with erythromycin, amoxicillin, cotrimoxazole, chloramphenicol, tetracycline, azithromycin and clindamycin [14]. Hence, current treatment guidelines discourage the empirical use of antibiotics due to unnecessary antibiotic exposure and drug resistance [15]. Additionally, due to lack of β-lactamase enzyme production by *S. pyogenes*, it was considered universally susceptible to penicillin group and later generation of β-lactam antibiotics. Even though, early untreated *S. pyogenes* acute pharyngitis leads to post infection complications such as acute rheumatic fever (ARF) and rheumatic heart disease (RHD) and glomerulonephritis [16].

There is not much information on the screening of children for carriage of *S. pyogenes* in Ethiopia [17] but empirical treatment is the current practice in the study area and at large in Ethiopia. Additionally, people do not complete their treatment or took irregularly. Even though all these practices might contribute for drug resistance emergency in the study area, the study on ` prevalence, AST and associated factors of *S. pyogenes* acute pharyngitis among pediatric patients was not done in Amhara region, Bahir Bar. Additionally, empirical treatment is common on acute pharyngitis without any laboratory identification of real pathogens. However, the clinical predictors of acute phayngitis were variable across geographical regions. Therefore, this study aimed to determine the prevalence, antibiotics susceptibility profiles and associated factors of *S. pyogenes* among pediatric patients with acute pharyngitis in FHCSH, Bahir Dar, Northwest Ethiopia.

## Methods

### Study design, period, area and population

Hospital based cross-sectional study was carried out from 1st February to 19th June 2020 at FHCSH in Bahir Dar, Ethiopia.**.** In FHCSH, there was 121 empirically treated acute pharyngitis cases in the last February to may, 2019 [18]. Padiatric patients attending to FHCSH with acute pharyngitis were the study population.

### Inclusion and exclusion criteria

All children with age ≤ 18 years and with symptoms of acute pharyngitis at FHCSH were included to this study. Whereas, those who took antibiotics within 2 weeks of sample collection were excluded in this study.

### Sample size determination and sampling technique

The sample size was calculated using a single population proportion formula based on the assumption of 5% expected margins of error, 95% confidence interval (Za/2 = 1.96) or alpha (α = 5%) and 11.3% prevalence based on a study in Jimma town, Ethiopia [11].

$$ \mathrm{N}=\frac{{\left(\mathrm{Za}/2\right)}^2\times \mathrm{P}\left(1-\mathrm{P}\right)}{{\mathrm{d}}^2}=\frac{(1.96)^2\times 0.113\left(1-0.113\right)}{\left({0.05}^2\right)}=154, $$

where p- prevalence

d- margin of error

N- Number of sample size

Thus, a total 154 throat samples were collected by consecutive convenient sampling technique.

### Data collection and processing

The data were collected by trained pediatric nurses and principal investigator. Thus, sociodemographic, environmental factors, behavioral and housing related data were collected by structured pre-tested Amharic version questionnaire using face to face interview with parents/ guardians. Participants whose age < 15 were interviewed via their gurdian and participants whose age > 15 years were interviewed directly after obtaining assent. Clinical data were collected by trained pediatric nurses. At each data collection spot, sufficient explanation about the aim of the research was given to the parents or study participants before conducting the interview.

### Sample collection and transportation

A single throat swab specimen was collected on the tonsils and the posterior pharynx from each study participants for culture using sterile cotton swab. The specimen could be collected at symptomatic area at which cotton swab rolled three times on exudates, inflamed pharynx, and swollen tonsil. Tongue depressor was used to depress tongue during throat swab collection [19]. Then, these throat swabs were transported using Amie’s transport media with cold box containing ice pack to Bahir Dar University, Microbiology Research Laboratory Center.

### *Streptococcus pyogenes* identification

Throat swabs were inoculated on 5% sheep blood agar plates and incubated at 37 °C in candle jar with 5% CO_2_ atmosphere for 24 h [20–22]*.* Catalase test was done from 24 h growth of β-hemolytic colonies to differentiate catalase negative *Streptococcus* species*.* This catalase negative *Streptococcus* species was subjected to bacitracin test. As such, colony suspension with normal saline matched with 0.5 McFarland standards was prepared from fresh 24 h growth of colonies and inoculated on 5% sheep blood agar. Then, imidiately placed bacitracin disk. Finally,, any inhibition was showed as bacitracin sensitive for *S. pyogenes* [20] after 24 h incubation at 37 °C in candle jar [19].

### Antibiotic susceptibility test

Antimicrobial susceptibility test (AST) was done by disk diffusion method on Mueller-Hinton (MHA) agar supplemented with 5% sheep blood [20]. Suspension was prepared from 3 to 5 pure *S. pyogenes* colonies mixed with 5 ml normal saline in sterile glass test tube which matched with 0.5 McFarland standards. Such suspension was evenly spread onto Mueller Hinton agar supplemented with 5% sheep blood using sterile cotton swab. The tested antibiotics included penicillin (*P* = 10 U), ampicillin (AMP = 10 μg), erythromycin (E = 15 μg), chloramphenicol (C = 30 μg), clindamycin (DA = 2 μg), tetracycline (TE = 30 μg), vancomycin (VA = 30 μg), levofloxacin (LEF = 5 μg), ceftriaxone (CRO = 30 μg), cefotaxime (CTX = 30 μg), Collect cefepime (FEP = 30 μg). After inoculation, it was incubated at 37^O^c in a candle jar for over 18 h. After then, zone of inhibition was measured with ruler and interpreted as sensitive, intermediate and resistant according to the principles established by CLSI M100 guideline [20].

### Quality control

The structured questionnaires were prepared in English and translated into Amharic language and then back translated to English to check inconsistencies of meaning of words. About 5% of structured questionnaire was pretested in Shegaw Motta General Hospital and training was also provided to pediatric nurse how to collect the data.

After throat swab samples were collected aseptically, transportation was carried out using Amies transport medium [22] to Bahir Dar university Microbiology Resaerch Laboratory Center by maintaining cold chain, or cold box with dry ice [23]. The culture media was prepared aseptically by autoclaving and the sterlity was checked by incubating 5% of the batch prepared media overnight. Additionally, the performance of the media was checked for growth of known *Streptococcus pneumoniae* (*S. pneumoniae*) ATCC 49619 as a positive control [20]. Likewise, Bacitracin test was checked by *Streptococcus pyogene* ATCC 19615 as positive and *Streptococcus agalactiae* ATCC 13813 as negative control.

### Data analysis

Data was entered by EpiData version 3.1 and data analysis was performed using SPSS version 20. The prevalence of *S. pyogenes* and antibiotics resistance was determined by descriptive statistics. Multivariable logistic regression was done by entering the variables with *p* < 0.2 in bivariable logistic regression to identify the associated factor and clinical predictors. A *P value <* 0.05 considered as statistically significant association in the multivariate logistic regression.

### Ethical consideration

The study approved by College of Medicine and Health Science, Bahir Dar University’s Research Institutional Review Board with reference number CMHS 0014/2020 and a permission letter was obtained from FHCSH. The purpose and importance of the study was explained to the participants. Written informed consent from parent/guardian and assent from children was obtained in accordance with the Declaration of Helsinki. Additionally, absence of link between the study and their service was explained and participation was entirely voluntary based. Furthermore, the confidentiality of study participant was kept and identification of study participant by name was avoided.

## Results

### Sociodemographic characteristics of study participants

A total of 154 pediatric children were recruited to this study. From those, the majority 81 (52.6%) of participants were females and 73 (47.4%) were males. Study participant’s age were ranged from 0 to 18 years old with mean age 8.483, median 9.0 and standard deviation (SD = 4.8). Majority of study participants were less than 5 years age 51 (33.1%) followed by 5–10 years of age 46 (29.9%) and 10-15 years of age 43 (27.9%). Moreover, about 102 (66.2%) participants were from urban. Additionally, the majority 65 (42.1%) of participants could not able to read & write (Table 1).

**Table 1:** Prevalence of *S. pyogenes* with respect to socio-demographic characteristics among pediatric patient with acute pharyngitis in FHCSH, Northwest Ethiopia, 1st February to 19th June 2020

Variables	Categories	Culture result for *S. pyogenes*		Total N (%)
		Positive N (%)	Negative N (%)	
Sex	Male	3 (2.0)	70 (45.45)	73 (47.45)
	Female	11 (7.1)	70 (45.45)	81 (52.55)
Age (in year)	< 5	0 (0)	51 (33.1)	51 (33.1)
	5–9	8 (5.2)	38 (24.7)	46 (29.9)
	10–14	5 (3.2)	38 (24.7)	43 (27.9)
	15–18	1 (0.7)	13 (8.4)	14 (9.1)
Residence	Urban	9 (5.9)	93 (60.4)	102 (66.3)
	Rural	5 (3.2)	47 (30.5)	52 (33.7)
Education level of children	Cannot read &write	5 (3.2)	60 (39.1)	65 (42.2)
	Can read & write	0 (0)	7 (4.7)	7 (4.7)
	Primary school	8 (5.2)	56 (34.4)	64 (41.6)
	Secondary school	1 (0.6)	16 (10.3)	17 (10.9)
	College and above	0 (0)	1 (0.6)	1 (0.6)
Occupation of parents/guardians	House wife	4 (2.65)	42 (27.3)	46 (29.95)
	Farmer	4 (2.65)	33 (21.4)	37 (24.05)
	Merchant	3 (1.9)	29 (18.8)	32 (20.7)
	Laborer	0 (0)	8 (5.2)	8 (5.2)
	Employed	3 (1.9)	28 (18.2)	31 (20.1)
Total		**14 (9.1)**	**140 (90.9)**	**154 (100)**

*N:* Frequency, *%* : Percent

### Prevalence of *Streptococcus pyogenes*

The overall prevalence of *S. pyogenes* was 14 (9.1%; 95%; CI = 4.5–14.3). Out of the total *S. pyogenes* culture positives with acute pharyngitis, females shared 11 (7.1%). According to age categories, there was no positive for *S. pyogenes* in the age less than 5 years. Whereas 8 (5.2%), 5 (3.2%) and 1 (0.7%) were age between 5 and 10, 10–15 and 15–18, respectively. Furthermore, about 8 (5.2%) *S. pyogenes* isolates were isolated from those participants in primary school. The isolation rates of *S. pyogenes* with different socio-demographic characteristics were summarized (Table 1).

### Antibiotics susceptibility profiles of *Streptococcus pyogenes*

**Fig. 1:**
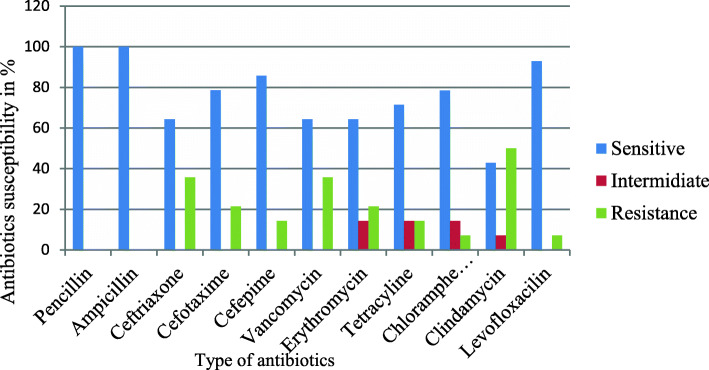
Antibiotic susceptibility profile of *S. pyogenes* isolates among pediatric patient with acute pharyngitis in FHCSH from 1st February to 19th June 2020

Different antibiotics classes were used for determining susceptibility profile of *S. pyogenes* isolates. As the result, all isolates of *S. pyogenes* were sensitive for both penicillin and ampicillin. Furthermore, the proportions of antibiotics resistances to clindamycin, ceftriaxone, cefotaxime, cefepime, vancomycin, erythromycin, tetracycline and chloramphenicol were 7 (50.0%), 5 (35.7%), 3 (21.4%), 2 (14.3%), 5 (35.7%), 3 (21.4%), 2 (14.3%) and 1 (7.1%), respectively. However, clindamycin 1 (7.1%), erythromycin 2 (14.3%), tetracycline 2 (14.3%) and chloramphenicol 2 (14.3%) were intermediate findings (Fig. 1).

Out of the total 14 *S. pyogene* isolates, the proportion of multidrug resistant isolates were 3(21.3%). From which, 1(7.1%) was multi drug resistance for erythromycin, clindamycin and vancomycin but 1(7.1%) for erythromycin, tetracycline and vancomycin. The rest 1(7.1%) was multidrug resistant for chloramphenicol, Cephame group and levofloxacin simultaneously.

### Factors associated with *Streptococcus pyogenes* acute pharyngitis

In univariables analysis, variables having a *P*-value of < 0.2 was entered in to multivariable logistic regression analysis. Based on this, sex, number of bed shared in home, frequency of cold drink, presence of smoker in home, separate kitchen and malnutrition were selected by forward likelihood logistic regression method. After adjusting other confounding variables, the pediatric children living with in the presence of any smokers in home showed 7.11 times more likely to develop *S. pyogenes* acute pharyngitis than those live in absence of any smokers in hone (AOR = 7.11, CI =1.694–29.82, *P* = 0.02) (Table 2).

**Table 2:** Bivariable and multivariable logistic regression analysis of factors associated with *S. pyogene*s acute pharyngitis in FHCSH, Northwest Ethiopia, 1st February to 19th June 2020

Variables	Categories	*S. pyogenes*		COR(95%CI) *P*- value	AOR(95%CI)*P*- value
		Pos (N)	Neg (N)		
Family number in home	> 5	13	75	11.2 (1.4–88.5)0.021*	–
	< 5	1	65	1	
Sex	Male	3	70	1	–
	Female	11	70	3.66 (2.98–13.71) 0.054*	
Age (in year)	< 5	0	51	3.92 (1.24–67.0) 0.35	–
	5–10	8	38	1.42 (1.05–3.72) 0.434	
	10–15	5	38	1.59 (1.06–5.450) 0.63	
	> 15	1	13	1	
Residence	Urban	9	93	1	–
	Rural	5	47	1.09 (0.35–3.47) 0.872	
Number of bed shared in home	> 2	2	72	6.35 (1.37–29.43) 0.018*	–
	< 2	12	68	1	
Frequently cold Drink	Yes	8	41	3.22 (1.05–9.86) 0.041*	–
	No	6	99	1	
Passive Smoker	Yes	8	30	4.89 (1.57–15.18) 0.006*	7.11 (1.69–29.82)0.01**
	No	6	110	1	1
Active smoker	yes	1	23	2.56 (1.32–20.51) 0.38	–
	No	13	117	1	
Separate kitchen	Yes	3	73	1.07 (1.01–3.55) 0.012*	2.05 (1.21–5.45) 0.16
	No	11	67	1	1
Malnutrition	Yes	1	4	2.12 (1.07–19.10) 0.06*	–
	No	13	136		

*N* Frequency, *COR* Crude odd Ratio, *AOR* Adjusted Odd Ratio, *Pos* Positive, *Neg* Negative, * = variable enrolled to multivariate regression (*P*- value < 0.2), ** = statistical significant

According to clinical predictors, all variables having a*P*-value< 0.2 in the bivariable was also subjected to multivariable analysis. Hence, the presence of tender lymphadenopathy were 14.45 times more likely to be *S. pyogenes* acute pharyngitis compared to those did not have tender lymphadenopathy (AOR = 14.45, 95% CI =1.6–30.3, *P* = 0.03). Similarly, the pediatric patients with history of recurrence were 5.87 time more likely to be acute pharyngitis caused by *S. pyogenes* compared to those did not having recurrence (AOR = 5.87, 95% CI = 1.63–12.31, *P* = 0.02). Therefore, tender lymphadenopathy and history of clinical recurrence (*P* < 0.05) were found to be independent predictors for *S. pyogenes* acute pharyngitis in pediatrics (Table 3).

**Table 3:** Bivariable and multivariable logistics regression analysis of chief clinical variables for *S. pyogenes* acute pharyngitis in FHCSH, Northwest Ethiopia, 1st February to 19th June 2020

Variables	Category	*S. pyogenes*		COR (95%CI) *P* value	AOR (95%CI) *P* value
		Pos (N)	Neg (N)		
Body temperature (In ^o^ C)	> 38	4	31	2.41 (1.41–8.79) 0.57	
	< 38	10	109	1	
Painful throat	Yes	13	121	2.04 (1.25–16.51) 0.50	
	No	1	19	1	
Headache	Yes	6	65	1.16 (1.08–3.50) 0.79	–
	No	8	75	1	
Vomiting	Yes	2	33	1.85 (1.94–8.69) 0.44	–
	No	12	107	1	
Abdominal pain	Yes	1	18	1.92 (1.24–15.56) 0.54	
	No	13	122	1	
Enlarged tonsil	Yes	8	75	1.86 (1.38–3.50) 0.80	–
	No	6	65	1	
Recurrence	Yes	11	54	5.84 (1.56–21.87) 0.01*	5.87 (1.89–26.78) 0.02**
	No	3	86	1	1
Inflame pharynx	Yes	9	87	1.10 (1.349–3.45) 0.88	–
	No	5	53	1	
Pharyngeal Exudate	Yes	12	84	4.0 (1.86–18.56) 0.08*	–
	No	2	56	1	
Lymphadenopathy	Yes	13	71	1.83 (1.6–5.56) 0.029*	14.45 (1.6–30.3) 0.02**
	No	1	69	1	1
Scarlatiniform Rash	Yes	10	41	12.6 (1.61–99.2) 0.02*	–
	No	4	99	1	
Dysphagia	Yes	10	79	1.93 (1.58–6.45) 0.29	–
	No	4	61	1	
Running nose	Yes	2	25	1.3 (1.28–6.20) 0.74	–
	No	12	115	1	

*N* frequency, *COR* Crude odd Ratio, *AOR* Adjusted Odd Ratio, *Pos* Positive, *Neg* Negative, * = variable entered to multivariate regression (*P*- value < 0.2), ** = statistical significant

## Discussion

*Streptococcus pyogenes* infection among the pediatric group is a cause of acute pharyngitis which could leads morbidity and mortality [24]. Pediatric patients with acute pharyngitis require microbiologic investigation and proper treatment to abort complications [25]. A totals of 154 throat swab samples were analyzed in the present study. Our 95% CI (4.5–14.3) used to clasfied studies in to low and high prevalence. For instance, prevalence studies with < 4.5% were considered as lower, and prevalence studies with > 14.3% is considered as higher findings and findings in between 95% CI of our study were considered as in agreement with current study.

Accordingly, the prevalence of *S. pyogenes* 14(9.1%) in the current study which is comparable with the previous conducted in Jimma, Ethiopia 11.3% [11], India 5.5% [16], Japan 5.8% [26], Indonesia 13.5% [27] and Nepal 9.2% [28]. But it was higher than a study from Mexico 0.04–0.42% [29], Brazil 3.9% [30], Romania 4% [31], Iran2.5% [32] and Saudi Arabia 1.5% [33]. This high prevalence rate in our study might be due seasonal nature of *S. pyogenes* incidence which is higher from February to May [11]. Additional reason for the difference might be sample size and method variation. In the contrary, the proportion of the recent study 9.1% was much lower than findings from USA 28.6–37% [34, 35] and 16–45% in African [36–39], Iran 30% [40] and Israel 69.5% [41]. Such variation could be attributed to difference geography, method, socio-economic conditions, and sample size.

Based on antibiotics susceptibility profiles in the present study, all isolates were sensitive to penicillin which is in agreement with studies reported in USA [29, 30], Asia [16, 42, 43], Europe [41, 44, 45], African countries including Egypt [46], Kenya [39] and Jima, Ethiopia [11]. This might be due to lack of β-lactamase production by *S. pyogenes*. Even though, penicillin resistance for *S. pyogenes* may happen by escaping penicillin treatment by entering epithelial cells, which are poorly penetrated by penicillin [47], by forming a biofilm [48] and protection of *S. pyogenes* by other β-lactamase-producing bacterial species [49, 50].

In accordance to other antibiotic resistance, simple comparison of resistance percentage was done comparatively, which was statistically higher or lower than those reported in the previous studies. Hence, it is known that erythromycin and clindamycin are usually used as an alternative treatment for patients allergic to penicillin. However, in the current study, erythromycin (21.4%) and clindamycin (50%) resistance were recorded which was higher than no resistant to erythromycin and clindamycin reported in Jimma, Ethiopia [11], erythromycin (10.6%) in Spain [45] but much lower than 64–83% resistance to erythromycin in India [43, 51]. Similarly, 35.7, 21.4 and 14.3% resistance to ceftriaxone, cefotaxime and cefepime were reported in our study which was higher than no resistance for ceftriaxone and cefotaxime in Pakistan [42] and India [16, 43] and Ethiopia [11], respectively. This variation might be due to physician provided treatment of non β-lactam drugs for acute pharyngitis case by considering Gram negative bacteria empirically. This might be the leading cause a high rate cephalosporin and erythromycin resistance. Additionally, erythromycin resistance in *S. pyogenes* occurs via target site modification that erythromycin ribosomal methylase (*erm*) genes encode an enzyme that methylate a single adenine in 23S rRNA and results conformational change in the ribosome, leading to reduced binding of erythromycin and clindamycin [52]. Similarly, in target drug efflux, *mefA* (macrolide efflux pump) genes encode an efflux pump of 14- and 15-carbonring macrolides, conferring resistance to erythromycin only. Furthermore, tetracycline resistance is conferred by ribosome protection genes such as *tet*(M) or *tet*(O) and efflux pumps for tetracycline encoded by the tet(K) or tet(L) gene also confer tetracycline resistance [53]. The erm and mefA genes are often collocated with tet gene of *S. pyogenes* strains are resistant to both macrolides and tetracycline [54].

According to factors in the current study, presence of any smokers in home (*P* value < 0.05) was associated with *S. pyogenes* acute pharyngitis in pediatric patients. This result was in agreement with previous finding reported in Northern India [44]. This might be due to presence of any smoker in home leads to children inhaled smokes which kills normal flora that able to compete pathogen from adherence, altered bacterial acquisition and make oral mucosal colonization in favor of *S. pyogenes* periodontal pathogens. Furthermore, the presence tender lymphadenopathy and recurrence (*P* value< 0.05) were found to be independent clinical predictors for *S. pyogenes* acute pharyngitis among pediatrics. Similar finding of tender lymphadenopathy and recurrences were reported in India [55], Yemen Saudi Arabia [56] and Jimma, Ethiopia [11]. However, clinical predictors varied with geographical area and immune status of study population [57].

### Limitation of the study

The limitations of this study were being small sample size which might be underestimating the prevalence of *S. pyogenes*. Additionally, MIC or E test was not done for better evaluation of its antibiotics susceptibility profiles.

## Conclusion

In this study, the prevalence of *S. pyogenes* in pediatric children with acute pharyngitis was 9.1%. All *S. pyogenes* remain sensitive to penicillin and ampicillin. The resistance rate was obtained to clindamycin 7 (50.0%), ceftriaxone 5 (35.7%), vancomycin 5 (35.7%), cefotaxime 3 (21.4%), erythromycin 3 (21.4%), cefepime 2 (14.3%) and tetracycline 2 (14.3%). The overall multidrug resistance was 21.3%. Relatively low resistance was documented to penicillin, ampicillin, levofloxacin and chloramphenicol. Hence, similar to other studies these drugs were considered as an empirical treatment for *S. pyogenes* acute pharyngitis in pediatric patients. The presence of any smoker in home was associated with *S. pyogenes* acute pharyngitis whereas tender lymphadenopathy and recurrence of sore throat were clinical predictors for *S. pyogenes* acute pharyngitis (*p* < 0.05) in our study. There should be routine throat culture and a continuous surveillance of antibiotics resistance pattern for *S. pyogenes* to improve the use of antibiotics in hospitals.

## Data Availability

The data supporting the conclusion of the study could be avaliabale upon the request of  Destaw Kebede (correspondence author; mobile + 251911594675, email: amaueldestaw@gmail.com).

## Abbreviations

ANRSHB: Amhara National Regional State Health Bureau
AOR: Adjusted Odd Ratio
APHI: Amhara Public health Institute
ARF: Acute Rheumatic Fever
AST: Antibiotic Susceptibility Test
ATCC: American Type Culture Collection
BAP: Blood agar plate
BDU: Bahir Dar University
CLSI: Clinical Laboratory of Standard Institute
CMHS: College of Medicine and Health Science
COR: Crude Odd Ratio
FHCSH: Felege Hiwot Comprehensive Specialized Hospital
GAS: Group A Streptococcus
IDSA: Infectious Diseases Society of America
ID: Individual identity
IQC: Internal Quality Control
MHA: Muller-Hinton agar
MIC: Minimum inhibitory concentration
OPD: Outpatient department
PYR: Pyrrolidonyl arylamidase test
RHD: Rheumatic heat disease
RADT: Rapid antigen detection test
SOPs: Standard operating procedures
SPSS: Statistical package for social sciences
URTI: Upper respiratory tract infection
US: United States
WHO: World Health Organization
